# The forgotten guidelines: cross-sectional analysis of participation in muscle strengthening and balance & co-ordination activities by adults and older adults in Scotland

**DOI:** 10.1186/s12889-016-3774-6

**Published:** 2016-10-21

**Authors:** Tessa Strain, Claire Fitzsimons, Paul Kelly, Nanette Mutrie

**Affiliations:** Physical Activity for Health Research Centre, Institute for Sport, Physical Education and Health Sciences, St Leonard’s Land, The University of Edinburgh, Holyrood Road, Edinburgh, EH8 8AQ UK

**Keywords:** Physical activity, Public health surveillance, Muscle strengthening, Balance, Co-ordination, Guidelines

## Abstract

**Background:**

In 2011, the UK physical activity guidelines were updated to include recommendations for muscle strengthening and balance & coordination (at least two sessions of relevant activities per week). However, monitoring and policy efforts remain focussed on aerobic activity. This study aimed to assess differences by gender and age in the a) prevalence of muscle strengthening and balance & co-ordination guidelines, and b) participation in guideline-specific activities.

**Methods:**

The sample for the muscle strengthening analyses was 10,488 adult (16–64 years) and 3857 older adult (≥65 years) 2012–2014 Scottish Health Survey respondents. The balance & co-ordination analyses used only the older adult responses. Differences by gender and (where possible) age in guideline prevalence and activity participation were assessed using logistic regression and t-tests.

**Results:**

Thirty-one percent of men and 24 % of women met the muscle strengthening guideline, approximately half that of published figures for aerobic physical activity. Nineteen percent of older men and 12 % of older women met the balance & co-ordination guidelines. The oldest age groups were less likely to meet both guidelines compared to the youngest age groups. Differences by gender were only evident for muscle strengthening: more men met the guidelines than women in all age groups, with the largest difference amongst 16–24 year olds (55 % men compared with 40 % women). Participation in relevant activities differed by gender for both guidelines. ‘Workout at gym’ was the most popular activity to improve muscle strength for men (18 % participated), while swimming was for women (15 % participated). Golf was the most popular activity to improve balance & co-ordination for older men (11 % participated) and aerobics was for older women (6 % participated). Participation decreased in most muscle strengthening activities for both men and women. One exception was golf, where participation levels were as high amongst older men as in younger age groups, although overall levels were low (3 % of all men).

**Conclusions:**

Physical activity policy should aim to increase prevalence of these ‘forgotten’ guidelines, particularly amongst young women (for muscle strengthening) and older age groups (both guidelines). Gender and age participation differences should be considered when designing population-level interventions.

**Electronic supplementary material:**

The online version of this article (doi:10.1186/s12889-016-3774-6) contains supplementary material, which is available to authorized users.

## Background

Increasing physical activity (PA) levels is a health priority in Scotland [1]. Progress is monitored by the proportion of the population undertaking the recommended amount of moderate and vigorous aerobic PA [2]. Until recently, the Scottish PA guidelines for adults focused only on aerobic activity. In 2011, the guidelines were updated to include recommendations on muscle strengthening (MS), balance & co-ordination (BC; for older adults (≥65 years) at risk of falls), and sedentary behaviour [3]. This paper focuses on MS and BC. The relevant additional recommendations are:

- Those over the age of 19 should undertake two sessions of MS activities per week, and
- Those over the age of 65 who are at risk of falls should undertake two sessions of BC activities per week [3].
  (see Table 1 for a list of the activities that were considered to improve MS and/or BC).

**Table 1:** Activities that are considered by the Scottish Health Survey to improve muscle strength and/or balance & co-ordination

Activity^*^	Muscle strengthening category	Balance & co-ordination category
Aerobics/Keep Fit/Gymanastics/Dance for fitness	b	a
Aquarobics/Aquafit/Exercise class in water	b	a
Athletics	a	a
Badminton/Tennis	b	a
Basketball	b	a
Canoeing/Kayaking	a	a
Climbing	a	a
Cricket	b	a
Curling	b	a
Cycling	b	a
Dancing (any other type)	b	a
Exercises	b	b
Fishing/angling	c	c
Football/Rugby	b	a
Golf	b	a
Hill walking/Rambling	b	a
Hockey	b	a
Horse riding	a	a
Ice skating	b	a
Powerboating/Jet skiing	c	a
Lawn Bowls	b	a
Martial arts/Tai Chi	b	a
Netball	b	a
Rowing	a	c
Running/Jogging	b	c
Sailing/Windsurfing	a	a
Shinty	b	a
Skateboarding/inline skating	c	c
Skiing/Snowboarding	a	a
Snooker/Billiards/Pool	c	c
Squash	b	a
Subaqua	c	c
Surf/Body boarding	b	a
Swimming	a	c
Table tennis	c	a
Tenpin bowling	b	a
Volleyball	b	a
Waterskiing	a	a
Workout at gym/Weight Training/Exercise bike	b	a
Yoga/Pilates	b	a

^*^The activities are listed as they are prompted in the Scottish Health Survey. No further details are available as to exactly what the respondent was referring to when they reported undertaking this activity

a) definitely a muscle strengthening and/or balance & co-ordination sport and exercise activity

b) only a muscle strengthening and/or balance & co-ordination sport and exercise activity if the respondent confirms in a follow up question (see text for more details)

c) not a muscle strengthening and/or balance & co-ordination sport and exercise activity

The inclusion of the MS guidelines for adults was in response to the growing evidence base showing that higher levels of muscle strength are associated with a reduced risk of premature mortality and cardiovascular disease across all ages, independent of aerobic PA levels [4–6]. There are also metabolic benefits to undertaking regular MS activities, such as improved insulin action, blood glucose control, and fat oxidation, all of which are critical in the prevention and treatment of type 2 diabetes and metabolic syndrome [7, 8]. There is tentative evidence to suggest MS activities improve self-esteem [9], and ameliorate symptoms of depression and anxiety [10, 11].

In older adults, MS activities limit the age-related decline in lean muscle mass (sarcopenia), help prevent osteoporosis, maintain functional capacity and reduce risk of falls [12–15]. Older adults may further reduce their risk of falls by undertaking BC activities [16, 17]. Studies in New Zealand and USA have found that around one-third of community-dwelling older adults fall each year [18, 19]. Considering the health and economic burden related to falls is high [14], this issue needs to be addressed.

In response to the additional guidelines, the Scottish national surveillance questionnaire (the Scottish Health Survey (SHeS)) was expanded so the MS activities of adults and older adults, and the BC activities of older adults could be monitored annually [20]. Whilst the SHeS records aerobic PA under the domains of walking, housework, heavy manual/Do-it-yourself home maintenance/gardening, occupational, and sport and exercise, designated MS and BC activities only appear within the sport and exercise domain [20] (full list in Table 1). Therefore, we use the terms MS and BC sport and exercise activities in this paper.

So far, the SHeS annual reports have only published descriptive statistics on the proportion of adults and older adults meeting the MS guidelines (27 % in 2012 [20]). There has been no statistical examination of the differences by age and gender, nor any analysis as to what MS sport and exercise activities adults and older adults undertake. There has been no analysis relating to the BC guidelines. This paper addresses these omissions by assessing whether there are any important and statistically significant differences by gender and (where possible given available bases) age group in:

i) the MS and BC guideline prevalence (and the proportions that undertaking no or insufficient activities)
ii) the participation levels in specific MS and BC sport and exercise activities.

This will provide a baseline from which progress can be monitored, suggest which activities are important in different sub-groups, and highlight sub-groups most in need of policy focus and intervention.

## Methods

### Data source

We obtained the 2012-2013-2014 SHeS combined dataset from the UK data archive on 17^th^ December 2015 [21]. The SHeS uses a two-stage stratified clustered sampling design to select households for participation in an interviewer-led computer assisted interview. After weighting, the data are nationally representative of the population living in private households in Scotland in 2012, 2013, and 2014. Further details on the sampling design and survey methods are in the SHeS Technical Report [22].

### Measurement of muscle strengthening and balance & co-ordination activities in the Scottish Health Survey

Adult respondents to the SHeS were asked to report the frequency (in the 28 days prior to interview) and average duration of any sport and exercise activities that they undertook. Over 40 sport and exercise activities were prompted and they were given the opportunity to report any others (for further details see Corbett et al. (2013) [23]). A panel of experts was convened to determine whether the prompted sport and exercise activities could count towards the MS and/or the BC guidelines [20]. Table 1 displays the three categories that they were allocated to: a) definitely a MS/BC sport and exercise activity, b) only a MS/BC sport and exercise activity if the respondent confirms in a follow up question, c) not a MS/BC sport and exercise activity. The follow up question for MS activities was “During the past four weeks, was the effort of (name of activity) usually enough to make your muscles feel some tension, shake or feel warm?” There was only one BC activity to require a follow-up question (exercises). The follow-up question to this activity was “Did these exercises involve you standing up and moving about?” The construct validity of this method has not been tested but we are unaware of any other validated method of assessing prevalence meeting national MS or the BC guidelines.

A respondent was deemed to have met the MS or the BC guidelines if they reported undertaking an average of ≥2 sessions of MS or BC sport and exercise activities respectively per week in the preceding 28 days. This is based on the assumption that the sessions took place on separate days. The UK PA guidelines do not specify a recommended bout length for MS or BC activities [3] and so the reported duration of activity was not taken into account. We calculated the proportions (1) achieving or exceeding these guidelines, (2) participating in some MS or BC sport and exercise activities but not sufficiently to meet the guidelines, or (3) not participating in any MS or BC sport and exercise activities.

Finally, we calculated the proportions that reported participating in each individual MS and BC sport and exercise activity in 28 days prior to interview. For category (b) activities where a follow up question was required to confirm that the activity was relevant, respondents only counted as participants if the answer was affirmative.

### Sample characteristics

There were 10,509 adult (16–64 years) and 3857 older adult (≥65 years) respondents to the 2012, 2013 and 2014 SHeSs. These were analysed together for the MS analyses. Those aged 16–18 were included in the analysis in line with UK health survey reporting although the PA guidelines defines adults as 19–64 years [3]. Only older adults were included in the BC analyses as the guideline only applies to this age group. It was not possible to identify those at risk of falls (the exact target group for the recommendation) and so we have analysed the data for all those over the age of 65.

Ten respondents were excluded from the MS analyses and one from the BC analyses as they did not answer the PA questions relating to sport and exercise. If there were missing data for a specific MS or BC sport and exercise activity, the respondent was kept in the overall analysis but that activity did not count towards the weekly total. Twelve further respondents were excluded from the MS analysis and one from the BC analysis as they averaged over 3 sessions per day for the previous 28 days. We considered these individuals as extreme outliers and not representative of normal populations. The MS analyses by age group used 10-year groups in line with standard health survey reporting; 5-year age groups were used for the BC analyses to provide further insight in the already restricted age range. Table 2 shows the unweighted and weighted sample sizes for the age and gender sub-groups (Table 2).

**Table 2:** The unweighted and weighted sample sizes for the age and gender sub-groups in the muscle strengthening and balance & co-ordination analyses

Muscle strengthening analyses								
	Age group							
	16–24	25–34	35–44	45–54	55–64	65–74	75+	Total
Men								
Unweighted	573	785	990	1165	1075	1063	669	6320
Weighted	970	1097	1101	1286	1071	812	535	6873
Women								
Unweighted	701	1082	1326	1469	1322	1180	945	8025
Weighted	966	1153	1169	1359	1125	908	780	7459
Balance & co-ordination analyses								
	Age group							
	65–69	70–74	75–79	80–84	85+	Total		
Men								
Unweighted	618	445	325	207	137	1732		
Weighted	487.3	325.2	260.1	160.2	114.6	1347		
Women								
Unweighted	679	500	437	301	207	2124		
Weighted	522.9	384.5	361.3	245	173.4	1687		

Note rows may not add up due to rounding

### Statistical analyses

Analyses were carried out using STATA/SE v14.1 using the ‘svyset’ commands to account for the design effects of the complex sampling strategy, following the recommendations of Heeringa et al. (2010) [24].

Multiple logistic regressions were performed on the proportions undertaking no, some, or sufficient MS or BC sport and exercise activities with the predictors age group, gender, and an interaction term. Significant differences compared to the reference category (youngest age group and males for the predictors respectively) were identified through Wald tests for the regression coefficients.

T-tests were performed to assess gender differences in the proportions taking part in the MS and BC sport and exercise activities (if the overall proportion participating was ≥1 %) using the ‘lincom’ command. Simple logistic regressions were used to test the differences in the proportions taking part in MS sport and exercise activities by age group, stratified by gender. Regressions were only undertaken if the activity featured in the top five for any age category for that gender. This was not possible for the BC sport and exercise activities as the sample sizes were too small.

A conservative Bonferroni adjusted α-level of 0.0003 was used to account for the large number of comparisons being made (184 test statistics). However, our conclusions have taken into account overall trends in the interpretation of the data and we comment only where differences appear to be of practical importance. One should be cautious interpreting these data based solely on this cut-off for statistical significance and therefore have provided the exact *p*-values and 95 % confidence intervals for the regression analyses in the Additional Tables (see Additional file 1).

## Results

### Muscle strength

**Fig. 1:**
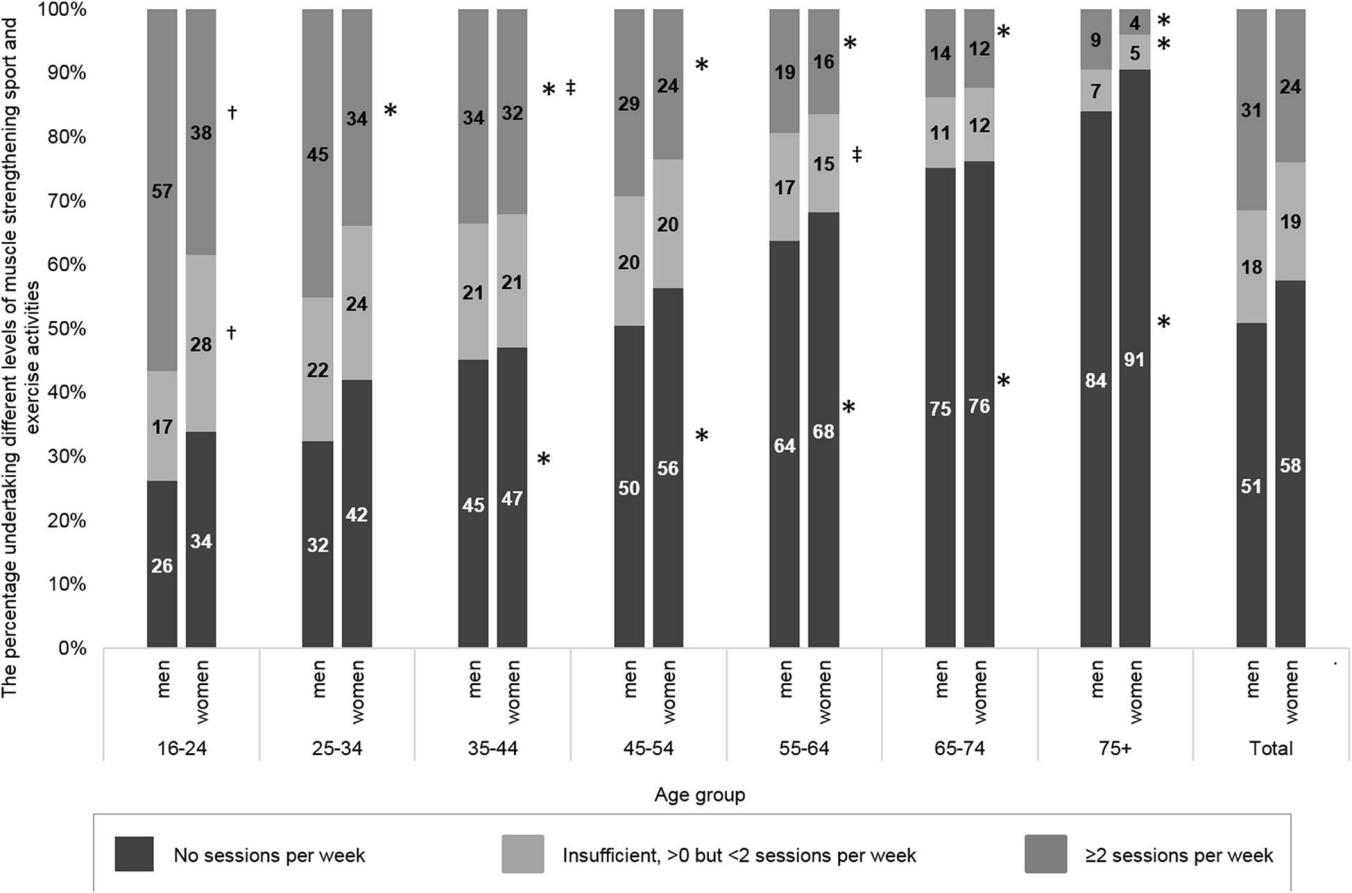
Levels of muscle strengthening sport and exercise activities, by age group and gender. Weighted *n* = 6873 men, *n* = 7459 women. *significantly different from 16 to 24 year age group at *p* < 0.0003. †significantly different between genders at *p* < 0.0003. ‡significant interaction between gender and age group at *p* < 0.0003

The proportions of men and women in Scotland in 2012–14 meeting the MS guidelines were 31 and 24 % respectively (Fig. 1, Additional file 1: Table S1). The proportions were highest amongst the youngest age group 16–24 year olds (57 % of males and 38 % of females); all other age groups were significantly less likely to meet the guidelines. The proportions decreased with age with the lowest amongst the over 75 s (9 % of men and 4 % of women in this age group). Men were more likely to meet the guidelines than women across all age groups, with the exception 35–44 year olds where the statistically significant interaction effect implied the 2 percentage point difference between the genders is with the range of variance.

The proportion doing some MS sport and exercise activities but at an insufficient frequency (>0 but <2 sessions per week over previous 28 days) to meet the guidelines ranged between 17 and 28 % for both genders between the ages of 16 and 54 years, before declining to 7 % for men and 5 % for women over 75 years. The difference between the youngest and oldest age groups was significant. Men were more likely to undertake some MS sport and exercise activities than women in the youngest age group (17 % for men and 28 % for women aged 16–24 years). Although the only interaction effect to meet our conservative α-level was for 55–64 year olds (implying no effect of gender in this age group), the difference between the genders was a maximum of two percentage points in all other (non-reference category) age groups.

The proportion undertaking no MS sport and exercise activities per week increased with age from 26 % of men and 34 % of women aged 16–24 to 84 % of men and 91 % of women over 75 years. This was significantly higher for those over the age of 35 compared with the youngest age group. There were no significant effects of gender, or interaction between gender and age group.

**Fig. 2:**
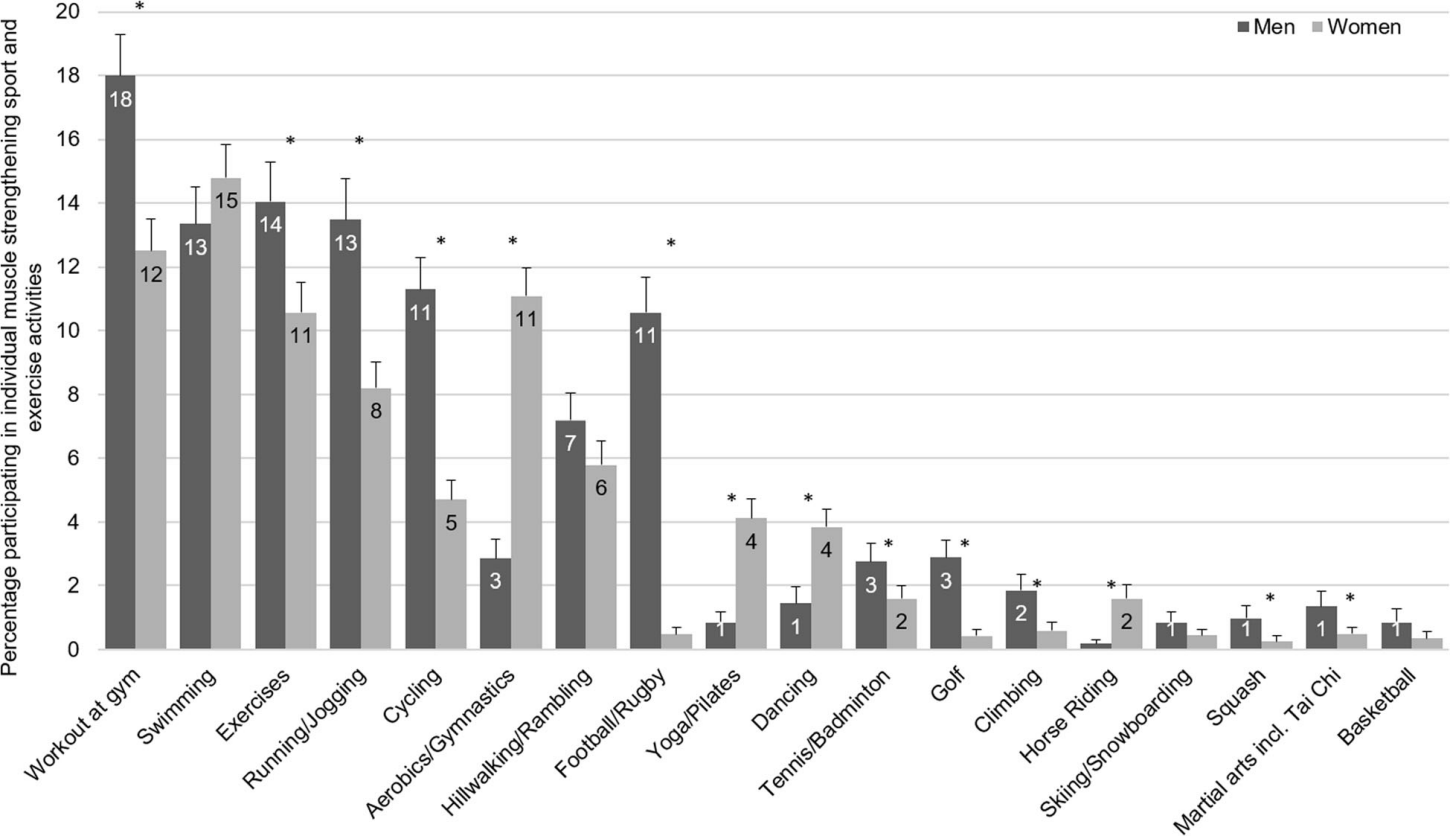
Participation in muscle strengthening sport and exercise activities, by gender. Weighted *n* = 6873 men, *n* = 7459 women. *significantly different between genders at *p* < 0.0003. Workout at gym includes ‘weight training’ and ‘exercise bike’. Aerobics/Gymnastics includes ‘keep fit’ and ‘dance for fitness’

Figure 2 shows the participation levels (at least 1 session in the previous 28 days) by gender for individual MS sport and exercise activities that had an overall prevalence ≥1 %. Men were more likely to participate in ‘workout at gym’ (including weight training and exercise bike), exercises, running/jogging, cycling, football/rugby, tennis/badminton, golf, climbing, squash, and martial arts (including tai chi); women were more likely to participate in aerobics/gymnastics, yoga/pilates, dancing, and horse-riding. The difference between the genders in participation levels was not significant for swimming, hill walking/rambling, skiing/snowboarding, and basketball.

Table 3 shows the proportion participating in the top five MS sport and exercise activities in each age group, stratified by gender (see also Additional file 1: Table S2). The proportions of both genders taking part in ‘workout at gym’ (including weight training and exercise bike), exercises, and running/jogging were significantly lower after the age of 35 compared to those aged 16–24. The decline was later in swimming for both genders; participation levels were significantly lower for men over 65 years and women over 55 years. Hill walking/rambling participation was maintained for both men and women up until the 65–74 age group. It decreased for both sexes amongst those over 75 years, although technically not significant for men at the conservative Bonferroni-adjusted α-level. Golf participation levels increased in the middle age groups for men, and there were similar participation levels amongst the youngest and oldest age groups. However, note that overall participation levels were low (3 % of all men, Fig. 2). Football and cycling participation levels were lower for men after the age of 25 and 55 respectively, compared to 16–24 year olds. Dancing participation declined for women after 35 years, whilst aerobics participation was significantly lower for women after the age of 55.

**Table 3:** Participation in the top five muscle strengthening activities, by age group, stratified by gender

Men													
16–24		25–34		35–44		45–54		55–64		65–74		75+	
Workout at gym	36	Workout at gym	29	Swimming	18	Workout at gym	16^*^	Swimming	10	Swimming	7^*^	Swimming	4^*^
Running	32	Running	25	Workout at gym	18^*^	Swimming	15	Workout at gym	9^*^	Workout at gym	6^*^	Workout at gym	3^*^
Football/Rugby	31	Exercises	24	Cycling	16	Cycling	15	Hillwalking	8	Hillwalking	5	Exercises	3^*^
Exercises	31	Swimming	19	Running	15^*^	Running	11^*^	Cycling	7^*^	Exercises	4^*^	Golf	3
Cycling	15	Football/Rugby	18^*^	Exercises	14^*^	Hillwalking	10	Exercises	7^*^	Golf	4^†^	Hillwalking	2
Women													
16–24		25–34		35–44		45–54		55–64		65–74		75+	
Workout at gym	24	Swimming	22	Swimming	20	Swimming	14	Swimming	12^*^	Swimming	8^*^	Aerobics	4^*^
Exercises	24	Workout at gym	20	Workout at gym	17^*^	Workout at gym	12^*^	Aerobics	7^*^	Aerobics	6^*^	Swimming	2^*^
Swimming	21	Aerobics	17	Aerobics	16	Aerobics	11	Workout at gym	6^*^	Workout at gym	4^*^	Exercises	1^*^
Running	18	Exercises	17	Exercises	14^*^	Exercises	8^*^	Hillwalking	6	Hillwalking	4	Dancing	1^*^
Aerobics	14	Running	16	Running	12^*^	Hillwalking	8	Exercises	4^*^	Exercises	3^*^	Hillwalking	1^*^

Weighted *n* = 6873 men, *n* = 7459 women

^*^significantly lower participation than 16–24 year age group at *p* < 0.0003

^†^significantly higher participation than 16–24 year age group at *p* < 0.0003. Workout at gym includes weight training and exercise bike. Aerobics includes ‘keep fit’, gymnastics and ‘dance for fitness’

### Balance & co-ordination

**Fig. 3:**
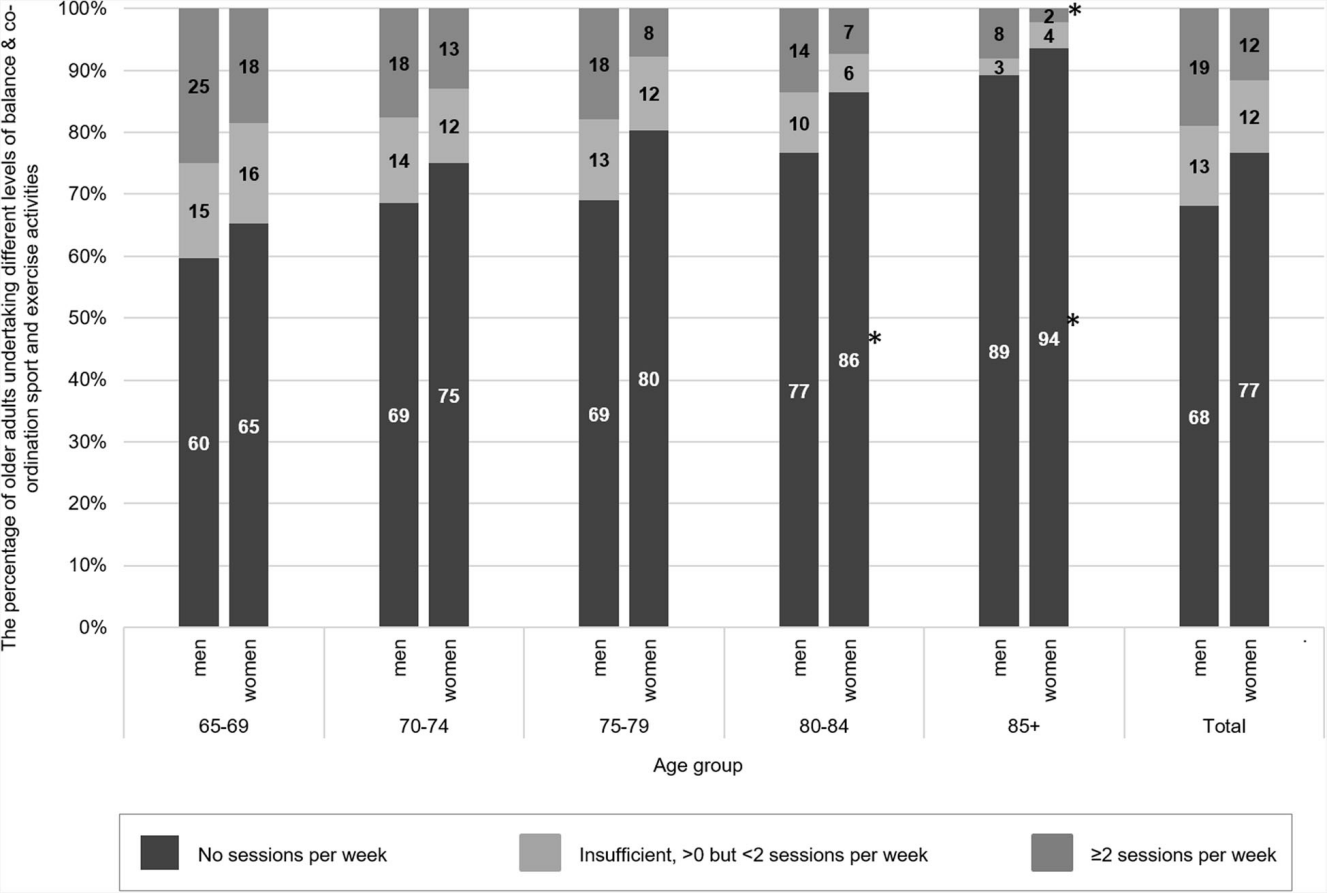
Levels of balance & co-ordination sport and exercise activities, by age group and gender. Weighted *n* = 1347 men, *n* = 1687 women. *significantly different from 16 to 24 year age group at *p* < 0.0003. †significantly different between genders at *p* < 0.0003. ‡significant interaction between gender and age group at *p* < 0.0003

The proportion of older adults meeting the BC guidelines in Scotland in 2012–14 was 19 % and 12 % for men and women respectively (Fig. 3, Additional file 1: Table S3). The proportion decreased steadily with age, from 25 % of men and 18 % of women aged 65–69 to 8 % of men and 2 % of women aged over 85 years, although the only significant difference was between the youngest and oldest age groups. There was no overall effect of gender nor any interaction effects.

The proportion undertaking some BC sport and exercise activities but at an insufficient frequency did not vary by age group or gender, ranging between 3 and 16 %. The proportion doing no BC sport and exercise activities increased with age from 60 % of men and 65 % of women aged 65–69 to 89 % of men and 94 % of women aged over 85 years. The proportions were significantly higher for those over the age of 80 compared to the youngest age group. There was no effect of gender or any interaction between gender and age group.

**Fig. 4:**
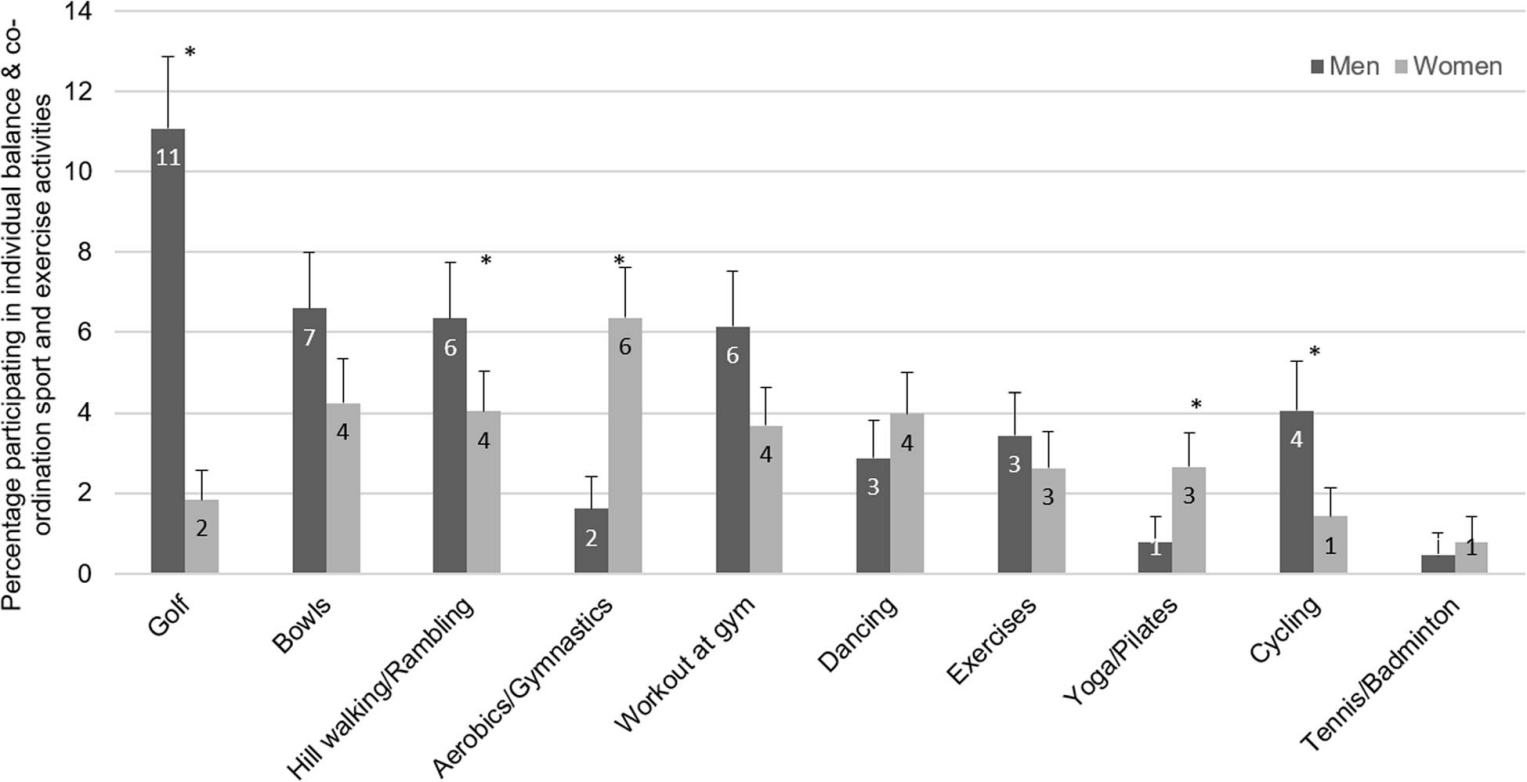
Participation in balance & co-ordination sport and exercise activities, by gender. Weighted *n* = 1347 men, *n* = 1687 women. *significantly different between genders at *p* < 0.0003. Workout at gym includes weight training and exercise bike. Aerobics/Gymnastics includes ‘keep fit’ and ‘dance for fitness’

Figure 4 shows that the participation levels for older adults were low across all BC sport and exercise activities. Golf was the most popular BC sport and exercise activity for men but it had the greatest difference between the genders (11 % of older men versus 2 % of older women). Aerobics/Gymnastics (including ‘keep fit’ and ‘dance for fitness’) was the most popular activity for older women with only 6 % taking part.

## Discussion

### Summary of findings

This is the first paper to provide detailed nationally representative information on the proportions meeting MS and BC guidelines amongst adults and older adults, by age and gender. We found that the oldest age groups were less likely to meet either guidelines compared to the youngest age groups. However, significant differences by gender were only evident for MS (more men met the guidelines than women across all age groups). Participation in individual MC or BC sport and exercise activities varied by gender and age group.

### Muscle strengthening

Approximately half as many adults and older adults in Scotland meet the MS guidelines (31 % of men and 24 % of women) compared to the aerobic PA guidelines (71 % men and 58 % of women [25]) in 2013. This calls into question whether the current focus on aerobic PA is appropriate particularly given the strong evidence demonstrating the health benefits of MS activities [4–6].

Few countries report nationally representative estimates for the proportion meeting the MS guidelines. Even amongst those that do measure relevant activities at a population level, there are large variations in the definitions and surveillance methods used, which may be obscuring or amplifying real differences. This is important to highlight given the number of inter-country PA comparisons that take place (e.g. GoPA! Country Cards [26], Active Healthy Kids Country Cards [27], the Global Burden of Disease studies [28]).

Within the UK there is a degree of consensus with both England and Northern Ireland using comparable methods to the SHeS [29, 30]. The proportions meeting the MS guidelines reported in this study are similar to those reported for England in 2012 (34 % of men and 24 % of women) [29] but are higher than Northern Ireland in 2013/14 (25 % of men and 14 % of women) [30]. In the USA, participants of the National Health Interview Survey are asked how often they do leisure-time physical activities specifically designed to strengthen their muscles such as lifting weights or doing calisthenics [31]. The 2014 survey estimated that 28 % of men and 20 % of women in the USA undertook a sufficient quantity of MS activities to meet the guidelines [32]. In Australia, different surveys use different methods and the estimates for the proportion of adults meeting the MS guidelines range between 9 and 19 % [33, 34].

Our findings highlight three key groups for policy focus and intervention. Firstly, promotion efforts should be focussed on women, particularly in the youngest age groups. We found the largest percentage point difference between the sexes was amongst 16–24 year olds (57 % compared with 38 %). This is concerning as bone and muscle mass peak in early adulthood and MS activities at this stage in life could help to maximise this and play a role in the prevention of osteoporosis. Both bone and muscle mass have been shown to decrease with age from the mid-20s, with an accelerated decline from age 50 onwards [13, 35]. This is apparent in both men and women, although hormonal changes associated with the menopause can further exacerbate the decline for women [13, 35]. Coupled with the fact that women, on average, have a smaller muscle mass than men, this means they tend to cross ‘thresholds for independence’ (the point at which a task cannot be completed independently) earlier [36].

Secondly, the proportions undertaking no MS sport and exercise activities over the age of 75 (84 % of men and 91 % of women) are concerning as muscle strength is of particular importance to older adults. One reason for this is because of the natural age-related decline of lean muscle mass (termed sarcopenia) [12]. Studies have estimated the decline to be around 2–4 % per year amongst those over 75 years, but the loss of strength can be 2–5 times faster than that because of other deleterious changes to muscle quality and neural factors [37]. This loss means that it can be muscle strength that is the primary limiting factor for functional independence [35], rather than aerobic PA. Low levels of muscle strength increase the risk of falling and sustaining a related injury, can lead to disability, and frailty [14, 38], all of which have implications for the individual, their carers, and the health services that support them. Strength training has been shown to be equally effective at increasing muscle strength in older adults as in younger adults, sometimes more so [39].

Thirdly, the 18 % of men and 19 % of women that undertook some but not a sufficient number of sessions of MS sport and exercise activities per week are targets where successful intervention may be more likely. If related to the trans-theoretical model, then these individuals could be considered to be in the ‘maintenance’ phase (i.e. already undertaking a relevant behaviour) [40]. It is potentially easier for them to increase the frequency of this behaviour to the recommended levels than for those not currently undertaking any to start.

The differences by gender and age of participation in MS sport and exercise activities are similar to the overall participation levels for sport and exercise activities in Scotland [41]. From this we can infer that, for those activities that require a follow up question to confirm they are a relevant activity, the responses do not vary greatly by age or gender. This suggests that efforts to narrow overall participation gaps go some way to reducing the inequalities in the prevalence of the MS guideline. Our results also highlight hill-walking (for both genders) and golf (for men) as two activities where participation levels are maintained in the older age groups. These are potentially important intervention activities as it has been shown that sustained participation in MS exercise, starting at a young age, provides the greatest protection against sarcopenia [42].

Although the UK PA guidelines for adults apply from aged 19 [3], we included 16–18 year olds in our analyses as this aligns with UK health survey reporting and provides more useful information to policymakers. We have undertaken a comprehensive sensitivity analysis: their inclusion makes a ≤1 percentage point difference to the proportions doing no, some and sufficient MS exercise amongst 16–24 year olds and does not change any overall conclusions. The UK guideline relating to MS for 5–18 year olds is combined with that for vigorous intensity aerobic activity: ‘Vigorous intensity activities, including those that strengthen muscle and bone, should be incorporated at least 3 days a week’ [3]. Given that, if anything, these MS guidelines are greater than for those ≥19 years, we do not feel that this is an unfair misrepresentation.

### Balance & co-ordination

We found that less than a fifth of older adults in Scotland (19 % of older men and 12 % of older women) met the BC guidelines in 2012–14. We found no differences in participation by gender, but a decline in the oldest two age groups. However, with such low levels of participation, we recommend that promotion efforts are aimed at all older adults rather than any specific groups.

Loss of the ability to balance is associated with a higher risk of falling and subsequent injury, which in turn can lead to loss of independence, illness, and premature mortality [43]. BC activities have been shown to be a critical part of an effective falls prevention programme [38]. One meta-analysis concluded that up to 42 % of falls could be prevented by a well-designed exercise programme that included BC activities [44].

Although the BC guideline applies to older adults at risk of falls [3], we included all older adults in our analyses as we were not able identify this ‘at risk’ group from the SHeS. This may have over- or under-estimated the proportions meeting the guideline. If those who are not at risk do not participate in any relevant activities then our estimates maybe lower than the true proportion. However, those who are not at risk may be more active, leading to an overestimation. We recommend that the target population of this guidelines is clarified, as this may hamper any co-ordinated effort to tackle the very low prevalence.

### Strengths and limitations

This study is the first to provide detailed analysis of the two forgotten guidelines: MS and BC. We have used routinely collected data to describe the current prevalence levels and identify key groups most in need of intervention. This is important information to take to policymakers to support the case for addressing these issues at a population level. Policy makers in Scotland use the results from national surveillance instruments to make decisions on funding and strategy [45]. Therefore it is appropriate to use these same data in this analysis, as it has most relevance for future policy decisions. The face validity of the SHeS method of measuring prevalence of population meeting the MS and BC guidelines is questionable as it is limited to sport and exercise activities. Although this is more inclusive than other national approaches to measuring MS that are often restricted to weight training or activities that would be categorised in the domain of sport and exercise [34, 46, 47]. Activities such as heavy gardening and carrying heavy loads are not included despite being listed as example activities in the guideline document itself [3]. Another limitation of the SHeS questionnaire is that certain activities are grouped together or cover a wide range of activities (e.g. workout at gym/weight training/exercise bike, or exercises) and it is not possible to establish which of the activities was undertaken and what exactly they involved.

As with all surveys, errors may arise at any stage: design, data collection, processing, and analysis [48]. One that is difficult to account for is the self-report nature of the data. It is possible that the reported levels of MS and BC activities differ from the true levels [49]. We add our support to calls to reach an international consensus over which activities should count towards the guidelines, how best to measure them at a population level [34], how to ensure they are of sufficient intensity, and then to investigate validation methods so that the degree of error can be better understood. Other factors such as sampling error or non-response bias are mitigated by the weighting procedures that result in a nationally representative sample on key demographic variables (see Bromley et al. (2015) for further details [22]). However, there remains a degree of uncertainty around the estimates and this should be considered in their interpretation.

## Conclusion

Our findings suggest that proportions meeting MS and BC guidelines are much lower than their aerobic counterpart. The promotion of PA should include efforts to increase the proportions meeting these forgotten guidelines. Particular efforts should be made amongst young women (for MS) and the older age groups (for MS and BC). Failure to do so could have important consequences as by 2031, the number of people over the age of 75 in Scotland is projected to rise by 75 % [50]. This will have implications for us as individuals and as a society if we do not change population levels of the many risk factors of ill health, of which strength and balance are two [4, 5, 16]. The most popular activities varied by gender and age and this should be considered when designing interventions. We also recommend further work on how best to monitor MS and BC activities at a population level.

## Additional file

Additional file 1: Table S1. The results from multiple logistic regression investigating the effect of age group, gender, and their interaction on participation levels of muscle strengthening (weighted *n* = 6873 men, 7459 women). **Table S2**: The results from simple logistic regression investigating the effect of age group on participation in specific muscle strengthening sport and exercise activities, stratified by gender (weighted *n* = 6873 men, 7459 women). **Table S3**. The results from multiple logistic regression investigating the effect of age group, gender, and their interaction on participation levels of balance & co-ordination (weighted *n* = 1347 men, 1687 women). (XLSX 29 kb)

## Abbreviations

BC: Balance & co-ordination
MS: Muscle strengthening
PA: Physical activity
SHeS: Scottish Health Survey
